# Bioaccumulation Factor of Selected Heavy Metals in Zea mays

**DOI:** 10.5696/2156-9614-9.24.191207

**Published:** 2019-12-06

**Authors:** Omolara Titilayo Aladesanmi, Jeremiah Gbenga Oroboade, Chisom Peter Osisiogu, Afolabi Olutope Osewole

**Affiliations:** Institute of Ecology and Environmental Studies, Obafemi Awolowo University, Ile-Ife, Nigeria

**Keywords:** heavy metals, maize, soil, plant, bioaccumulation factor

## Abstract

**Background.:**

Health risks arising from heavy metal pollution have attracted global attention. As a result, many studies on the accumulation of heavy metals in soil-plant systems have performed human health risk assessments.

**Objectives.:**

We aimed to examine the ability of Zea mays (maize) to accumulate heavy metals and assess the bioaccumulation factor (BAF) by collecting, collating, and analyzing data on heavy metal concentrations in Zea mays.

**Methods.:**

This study reviewed the accumulation of five selected heavy metals, cadmium (Cd), chromium (Cr), lead (Pb), copper (Cu), and zinc (Zn) in soil and the corresponding BAF of Zea mays grown on those soils using a systematic search of peer-reviewed scientific journals. A total of 27 research works were reviewed after screening 52 articles for subject matter relevancy, including dumpsites, industrially polluted soils, inorganically fertilized soils, mining sites, smelting sites, municipal wastewater irrigated soils, and a battery waste dumpsite.

**Results.:**

Among the reviewed sites, concentrations of Cd and Cr were highest at a tin mining site, where prolonged mining, mineral processing and other production activities contributed heavy metal pollution in the soil. The soil at a battery waste dumpsite exhibited the highest Pb concentration, while the soil at a Zn smelting site presented the highest concentration of Zn. The highest soil Cu concentration was found in an area where sewage irrigation had been carried out over a long period. The BAF of the five heavy metals in Zea mays increased with the metal concentrations in the soil. The BAF of Cd, Cr, Pb, Cu, and Zn in Zea mays from the study areas fall within the ranges of 0–0.95, 0–1.89, 0–1.20, 0.011–0.99, and 0.03–0.99, respectively. Cadmium and Zn had the highest bioconcentration factors values in maize plants, likely due to their higher mobility rate compared to the other heavy metals.

**Conclusions.:**

The study concluded that Zea mays is capable of accumulating high amounts of heavy metals, although accumulation of these heavy metals is influenced by multiple factors including soil texture, cation exchange capacity, root exudation and especially soil pH and chemical forms of the heavy metals. Zea mays should not be planted on metal-contaminated soils because of its potential to act as a hyperaccumulator.

**Competing Interests.:**

The authors declare no competing financial interests.

## Introduction

Heavy metal pollution is an environmental issue that has become a global problem. The production and emission of heavy metals has increased along with increased industrial development. This has led to increasing concern over food safety due to soil polluted with anthropogenic heavy metals released from industry or agriculture, such as smelting industries, residues from metalliferous mines, pesticides, fertilizers, and municipal composts.^1–14^

Heavy metals are chemical elements that have a relatively high density, strong toxic effects and pose an environmental threat.^15^ Heavy metals are of considerable environmental concern due to their toxicity, many sources, non-biodegradable properties, and accumulative behaviors.^16^ The presence of heavy metals in foods poses serious health hazards, depending on their relative levels. The ability of plants to accumulate metals and possibly other contaminants varies with both the nature of the plant species and the nature of the metal contaminant. Cereals, in this case Zea mays L (maize), are known to be good accumulators of contaminants.^17^

Agricultural soils in many parts of the world are slightly to moderately contaminated by heavy metal toxicity, such as cadmium (Cd), copper (Cu), zinc (Zn), nickel (Ni), cobalt (Co), chromium (Cr), lead (Pb), and arsenic (As). This could be due to longterm use of phosphatic fertilizers, sewage sludge application, dust horn smelters, industrial waste and poor watering practices in agricultural lands.^18–20^ The primary response of plants is the generation of reactive oxygen species upon exposure to high levels of heavy metals. Various metals either generate reactive oxygen species directly through Haber-Weiss reactions or overproduce reactive oxygen species and the occurrence of oxidative stress in plants could be the indirect consequence of heavy metal toxicity.^21^,^22^ The indirect mechanisms include their interactions with the antioxidant system, disrupting the electron transport chain or disturbing the metabolism of essential elements.^23–25^ One of the most deleterious effects induced by heavy metals exposure in plants is lipid peroxidation, which can directly cause biomembrane deterioration. Malondialdehyde, one of the decomposition products of polyunsaturated fatty acids of a membrane, is regarded as a reliable indicator of oxidative stress.^26^ Such toxic elements are considered soil pollutants due to their widespread occurrence, and their acute and chronic toxic effect on plants grown.

Zea mays L. is an annual cereal plant of the Gramineae family native to Mexico, it is one of the oldest and widely cultivated cereals and serves as food for humans and feed for livestock.^27^,^28^ Zea mays L. has been a major food source for humans since ancient times. It is a domesticated plant and has many beneficial uses for people and animals. Maize is one of the most intensively cultivated cereals worldwide. It is a basic staple food grain for large parts of world and is the main food energy source in developing countries, including Africa, Latin America, and Asia.^29^,^30^ In Nigeria, maize can be found in every city and village where it is consumed as a staple food.^28^ Zea mays L. is the third most important cereal grain worldwide, after wheat and rice.^31^ All parts of the crop can be used for food and non-food products, it can also be used in animal feed as a feedstock source in agricultural complexes.^32^ In industrialized countries, maize is largely used as livestock feed and as raw material for industrial production. It contains the vitamins A, B, C and E, including mineral salts and essential trace elements such as carotene, thiamine, ascorbic acid and tocopherol.^33^ Zea mays L. is a widely cropped annual cereal that grows rapidly, produces extensive fibrous root systems with large shoot biomass yield per hectare, withstands adverse conditions, and produces abundant seeds with ease of cultivation under repeated cropping. To date, over 400 taxa of plant hyperaccumulators of heavy metals have been identified, but most of them are low biomass producers and exotic species. There is a need to supplement the list of plants available for phytoextraction. The potential use of maize, a robust tropical cereal crop in phytoextraction technology and possible utilization of the by-product is especially advocated for developing countries with scarce funds available for environmental restoration.

**Abbreviations**
*BAF*: Bioaccumulation factor
*t*: Test statistic
*WHO*: World Health Organization

Several studies on the effects of bioaccumulation in plants through uptake of heavy metals from soils at high concentrations have been carried out and indicate great health risks, taking into consideration food chain implications. Utilization of food crops contaminated with heavy metals is a major food chain route for human exposure, especially those under continuous cultivation. The cultivation of such plants in contaminated soil represents a potential risk since the vegetal tissues can accumulate heavy metals.^34^ Heavy metals become toxic when they are not metabolized by the body and accumulate in soft tissues.^35^ Chronic ingestion of toxic metals has undesirable impacts on humans and the associated harmful impacts become perceptible only after several years of exposure.^36^

The bioaccumulation factor (BAF) is used to quantify the bioaccumulation effect of maize in the uptake of heavy metal from soil. The BAF evaluates the effectiveness of a plant in metal accumulation and translocation.^37^

Heavy metal contaminants not only represent a threat to agricultural product safety, but also harm the immune, reproductive, and nervous systems of organisms after entering their bodies through ingestion. Therefore, the transfer and accumulation of heavy metals in soil-plant systems has become an important research topic. We aimed to examine the ability of Zea mays to accumulate heavy metals and assess the bioaccumulation factor (BAF) by collecting, collating, and analysing data on heavy metal concentrations in Zea mays.

## Methods

Data related to the research topics was collected from published journals, articles, textbooks and dissertations sourced from PubMed, Google Scholar, JSTOR, Science Direct and AJOL.

### Methodology flow chart

**Figure 1:**
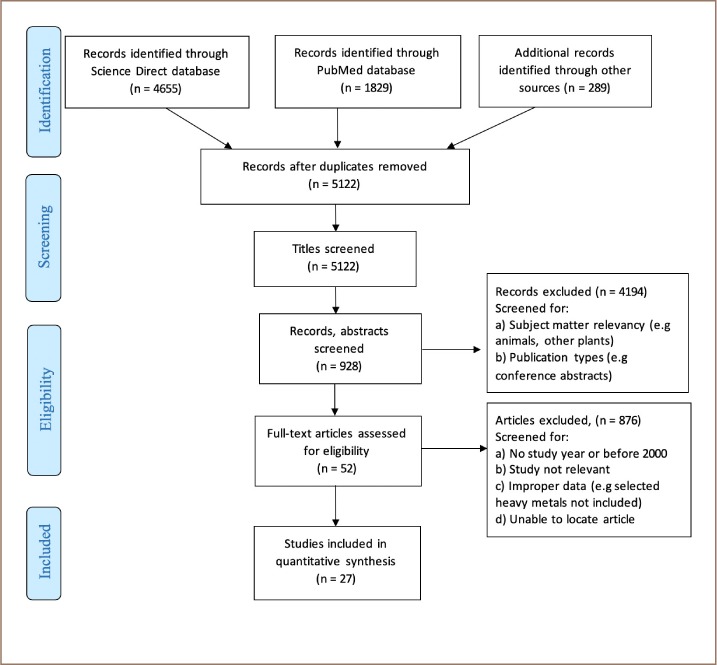
PRISMA flow diagram indicating the articles collection, screening exclusion and inclusion process

A Preferred Reporting Items for Systematic Reviews and Meta-Analyses (PRISMA) flow diagram indicating the number of articles that were identified, screened, and included in the current review is shown in Figure 1. Search terms were heavy metals, Zea mays, and bioaccumulation.

### Determination of bioaccumulation factor

The BAF is the ratio of the concentration of heavy metals in plants and in soils. It is an indicator of a plant's capacity to accumulate heavy metals.^38^

The BAF was calculated using Equation 1:

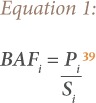
where, P_i_ is the concentration of a heavy metal in plants (mg/kg^−1^); and S_i_ is the concentration of the same heavy metal in the soil where the plant grows (mg/kg^−1^).

BAF was provided in the following articles shown in Table 1.

**Table 1:** Included Articles with Bioaccumulation Factors

**S/N**	**AUTHORS**	**YEAR**
1	Oladejo *et al.,*	2017^41^
2	Yang *et al.,*	2013^44^
3	Yu *et al.,*	2017^51^
4	Asgari and Cornelis	2015^56^
5	Afolayan and Hassan	2017^58^
6	Malomo *et al.,*	2013^61^
7	Lu *et al.,*	2015^62^

However, Equation 1 was applied in the articles presented in Table 2 where the P_i_ (concentration of heavy metal in Zea mays) and S_i_ (concentration of the same heavy metal in the soil) was provided.

**Table 2:** Included Articles Where Equation 1 was Applied

**S/N**	**AUTHORS**	**YEAR**
1	Awokunmi *et al.,*	2014^40^
2	Stanislawska–Glubiak *et al.,*	2015^42^
3	Prabpai *et al..*	2009^43^
4	Cai *et al.,*	2014^45^
5	Li *et al.,*	2008^46^
6	Jin *et al.,*	2014^47^
7	Zhu	2013^48^
8	Kang *et al.,*	2011^49^
9	Wang *et al.,*	2008^50^
10	Nan *et al.,*	2002^52^
11	Bi *et al.,*	2006^53^
12	Bi *et al.,*	2009^54^
13	Nwite and Alu	2015^55^
14	Liu *et al.,*	2005^57^
15	Ibrahim *et al.,*	2015^59^
16	Mantovia *et al.,*	2005^60^
17	Mu *etal..*	2013^63^
18	Alushllari *et al.,*	2013^64^
19	Rattan *et al.,*	2005^65^
20	Zojaji *et al.,*	2014^66^

### Statistical analysis

Data retrieved were analyzed on the stated hypothesis using descriptive statistics. Descriptive statistics were used to analyze and perform a one-sample Student's t-test to test the hypothesis that the mean concentration of each heavy metal in Zea mays is not significantly different from the permissible limit set by the World Health Organization (WHO).

## Results

Bioaccumulation of heavy metals, including Cd, Cr, Pb, Cu and Zn in Zea mays has been established by several studies worldwide using different indicators and parameters. Tables 3–7 show the bioaccumulation of selected heavy metals in Zea mays. Tables 8–12 show the BAF of soils across different sites and studies. The BAFs of Cd, Pb, Cr, Zn, and Cu in Zea mays from the study areas fall within the ranges of 0–0.95, 0–1.20, 0–1.89, 0.03–0.99, and 0.011–0.99, for dumpsites, industrially polluted soil, mining and smelting sites, municipal waste water irrigated soils and a battery waste dumpsite, respectively.

**Table 3:** Cadmium Concentration in Soil and Bioaccumulation Factors in Zea mays

**Reference**	**Number of samples**	**Type ofsite**	**Cadmium (mg kg^−1^)**	**Bioaccumulation factor**
Awokunmi *et al.* 2014^40^	40	a	21.9–138	0.03–0.058
Oladejo *et al.* 2017^41^	12	a	1.40–2.59	0.15–0.44
Stanislawska–Glubiak *et al.* 2015^42^	16	b	0.09–0.29	0.17–0.56
Prabpai *et al.* 2009^43^	20	b	0.05–1.69	0.01–0.12
Yang *et al.* 2013^44^	17	b	0.14–0.24	0.1–0.25
Cai *et al.* 2014^45^	27	b	0.11–3.11	0.00076–0.0049
Li *et al.* 2008^46^	30	b	0.81–1.5	0.13–0.74
Jin *et al.* 2014^47^	27	b	1.58–3.87	0.0019–0.002
Zhu 2013^48^	16	b	0.13–0.17	0.01–0.07
Kang *et al.* 2011^49^	15	b	0.142–0.162	0.0081–0.051
Wang *et al.* 2008^50^	12	b	0.11–3.99	0.16–0.43
Yu *et al.* 2017^51^	55	b	0.119–0.199	0.081–0.135
Nan *et al.* 2002^52^	33	c	0.14–19.3	0.063–0.95
Bi *et al.* 2006^53^	15	c	5.8–74.0	0.01–0.54
Bi *et al.* 2009^54^	55	c	69.0–2300.0	0.005–0.59
Nwite and Alu 2015^55^	27	d	10.03–10.56	0.0028–0.003
Asgari and Cornelis 2015^56^	96	d	3.8–4.1	0.05–0.28
Liu *et al.* 2005^57^	24	d	0.1–0.27	0.47–0.71
Afolayan and Hassan 2017^58^	17	e	163.96–258.38	0.176–0.197

Abbreviations: a, dumpsites; b, industrial pollution; c, mining and smelting; d, municipal water irrigated; e, battery waste dumpsite.

**Table 4:** Lead Concentration in Soil and Bioaccumulation Factors in Zea mays

**Reference**	**Number of samples**	**Type of site**	**Lead (mg kg^−1^)**	**Bioaccumulation factor**
Awokunmi *et al.* 2014^40^	40	a	35.0–60.0	0.24–1.20
Oladejo *et al.* 2017^41^	12	a	6.36–7.76	0.06–0.32
Ibrahim *et al.* 2015^59^	9	b	12.73–32.40	0.49–1.08
Mantovia *et al.* 2005^60^	12	b	15.7–15.8	0.006–0.007
Stanislawska–Glubiak *et al.* 2015^42^	16	b	21.7–34	0.01–0.013
Prabpai *et al.* 2009^43^	20	b	4.1–83.8	0.001–0.017
Cai *et al.* 2014^45^	27	b	139.10–651.97	0.00048–0.0025
Jin *et al.* 2014^47^	27	b	33.62–122.1	0.007–0.009
Zhu 2013^48^	16	b	20.68–28.65	0.00077–0.0016
Kang *et al.* 2011^49^	15	b	16.8–18.4	1.97E-6–4.64E-6
Wang *et al.* 2008^50^	12	b	11.2–29.97	0.0037–0.011
Yu *et al.* 2017^51^	55	b	34.42–42.27	0.0008–0.001
Malomo *et al.* 2013^61^	24	b	83.3–177.5	0.679–0.922
Bi *et al.* 2006^53^	15	c	60.0–570.0	0.002–0.119
Bi *et al.* 2009^54^	55	c	7.4–55	0.083–0.909
Nwite and Alu 2015^55^	27	d	34.76–39.75	0.00075–0.0086
Lu *et al.* 2015^62^	40	d	10.89–12.39	0.11–0.19
Liu *et al.* 2005^57^	24	d	13.0–24.5	0.14–0.19
Afolayan and Hassan 2017^58^	17	e	3265.8–1273.8	0.0096–0.0105

Abbreviations: a, dumpsites; b, industrial pollution; c, mining and smelting; d, municipal water irrigated; e, battery waste dumpsite.

**Table 5:** Chromium Concentration in Soil and Bioaccumulation Factors in Zea mays

**Reference**	**Number of samples**	**Type of site**	**Chromium (mg kg^−1^)**	**Bioaccumulation factor**
Awokunmi *et al.* 2014^40^	40	a	9.0–29.8	0.46–1.89
Oladejo *et al.* 2017^41^	12	a	7.92–10.99	0.14–0.41
Cai *et al.* 2014^45^	27	b	149.4–170.19	0.0011–0.015
Li *et al.* 2008^46^	30	b	51.0–69.0	0.0012–0.0015
Kang *et al.* 2011^49^	15	b	54.6–69.4	0.0039–0.0066
Wang *et al.* 2008^50^	12	b	38.4–55.8	0.011–0.019
Yu *et al.* 2017^51^	55	b	56.51–65.61	0.01–0.012
Zhu 2013^48^	16	b	58.73–62.18	0.0037–0.0057
Bi *et al.* 2006^53^	15	b	71.0–240.0	0.004–0.056
Prabpai *et al.* 2009^43^	20	b	7.1–23.8	0.0025–0.01
Mantovia *et al.* 2005^60^	12	c	58.3–59.6	0.013–0.015
Zojaji *et al.* 2014^66^	12	d	11.15–26.68	0.11–0.40
Asgari and Cornelis 2015^56^	96	d	23.8–45.3	0.01–0.12
Lu *et al.* 2015^62^	40	d	11.12–12.05	0.45–1.03
Liu *et al.* 2005^57^	24	d	49.0–162.0	0.04–0.08

Abbreviations: a, dumpsites; b, industrial pollution; c, mining and smelting; d, municipal water irrigated.

**Table 6:** Zinc Concentration in Soil and Bioaccumulation Factors in Zea mays

**Reference**	**Number of samples**	**Type of site**	**Zinc(mg kg^−1^)**	**Bioaccumulation factor**
Awokunmi *et al.* 2014^40^	40	a	63.0–80.2	0.07–0.4
Oladejo *et al.* 2017^41^	12	a	156.78–243.81	0.047–0.4
Ibrahim *et al.* 2015^59^	9	b	27.21–30.78	0.23–0.99
Stanislawska–Glubiak *et al.* 2015^42^	16	b	95.0–165.0	0.18–0.4
Mu *et al.* 2013^63^	18	b	80.6–108.9	0.20–0.26
Alushllari *et al.* 2013^64^	15	b	54.0–102.0	0.14–0.53
Yu *et al.* 2017^51^	55	b	70.58–85.15	0.203–0.245
Mantovia *et al.* 2005^60^	12	c	82.8–93.9	0.34–0.43
Nan *et al.* 2002^52^	33	c	43.5–565.0	0.189–0.73
Bi *et al.* 2006^53^	15	c	260.0–5500.0	0.03–0.25
Rattan *et al.* 2005^65^	115	d	99.68–99.86	0.678–0.789
Asgari and Cornelis 2015^56^	96	d	146.1–238.9	0.18–0.34
Lu *et al.* 2015^62^	40	d	8.47–12.13	0.1–0.26
Liu *et al.* 2005^57^	24	d	16.0–162.5	0.44–0.88

Abbreviations: a, dumpsites; b, industrial pollution; c, mining and smelting; d, municipal water irrigated.

**Table 7:** Copper Concentration in Soil and Bioaccumulation Factors in Zea mays

**Reference**	**Number of samples**	**Type of site**	**Copper (mg kg^−1^)**	**Bioaccumuiation factor**
Awokunmi *et al.* 2014^40^	40	a	7.0–18.0	0.44–0.68
Oladejo *et al.* 2017^41^	12	a	20.17–21.41	0.1–0.47
Prabpai *et al.* 2009^43^	20	b	7.0–82.1	0.065–0.66
*Yu et al.* 2017^51^	55	b	19.21–22.63	0.056–0.066
Malomo *et al.* 2013^61^	24	b	4.0–12.2	0.65–0.94
Alushllari *et al.* 2013^64^	15	b	16.6–21.1	0.07–0.42
Bi *et al.* 2006^53^	15	b	9.3–260.0	0.015–0.16
Mu *et al.* 2013^63^	18	b	30.8–36.3	0.57–0.65
Mantovia *et al.* 2005^60^	12	c	60.7–65.7	0.031–0.035
Ibrahim *et al.* 2015^59^	9	d	2.25–33.97	0.011–0.99
Rattan *et al.* 2005^65^	115	d	99.91–99.94	0.133–0.149
Asgari and Cornelis 2015^56^	96	d	51.9–64.3	0.06–0.12
Liu *et al.* 2005^57^	24	d	13.5–88.0	0.27–0.86

Abbreviations: a, dumpsites; b, industrial pollution; c, mining and smelting; d, municipal water irrigated.

**Table 8:** Cadmium Concentrations in Soil and Bioaccumulation Factors in Zea mays Based on Site Type

**Type of site**	**Reference**	**Cadmium (mg kg^−1^)**	**Bioaccumulation factor**
Dumpsite	Oladejo *et al.* 2017^41^	1.40–2.59	0.15–0.44
	Awokunmi *et al.* 2014^40^	21.9–138	0.03–0.058
Industrial pollution and inorganic fertilizer	Yu *et al.* 2017^51^	0.119–0.199	0.081–0.135
Mining and smelting	Nan *et al.* 2001^52^	0.14–19.3	0.063–0.95
	Bi *et al.* 2006^53^	69.0–2300.0	0.005–0.59
	Bi *et al.* 2009^54^	0.14–0.24	0.1–0.25
Municipal wastewater irrigated	Liu *et al.* 2005^57^	0.1–0.27	0.47–0.71
	Asgari and Cornelis 2015^56^	3.8–4.1	0.05–0.28
Battery waste dumpsite	Afolayan and Hassan 2017^58^	163.96–258.38	0.176–0.197

**Table 9:** Lead Concentrations in Soil and Bioaccumulation Factors in Zea mays Based on Site Type

**Type of site**	**Reference**	**Lead (mg kg^−1^)**	**Bioaccumulation factor**
Dumpsite	Oladejo *et al.* 2017^41^	6.36–7.76	0.06–0.32
	Awokunmi *et al.* 2014^40^	35.0–60.0	0.24–1.20
Industrial pollution and inorganic fertilizer	Malomo *et al.* 2013^61^	83.3–177.5	0.679–0.922
	Yu *et al.* 2017^51^	34.42–42.27	0.0008–0.001
	Ibrahim *et al.* 2015^59^	12.73–32.40	0.49–1.08
Mining and smelting	Bi *et al.* 2006^53^	60.0–570.0	0.002–0.119
	Bi *et al.* 2009^54^	7.4–55.0	0.083–0.909
Municipal wastewater irrigated	Liu *et al.* 2005^57^	13.0–24.5	0.14–0.19
	Lu *et al.* 2015^62^	10.89–12.39	0.11–0.19
Battery waste dumpsite	Afolayan and Hassan 2017^58^	3265.8–4273.8	0.0096–0.0105

**Table 10:** Chromium Concentrations in Soil and Bioaccumulation Factors in Zea mays Based on Site Type

**Type of site**	**Reference**	**Chromium (mg kg^−1^)**	**Bioaccumulation factor**
Dumpsite	Oladejo *et al.* 2017^41^	7.92–10.99	0.14–0.41
	Awokunmi *et al.* 2014^40^	9.0–29.8	0.46–1.89
Industrial pollution and inorganic fertilizer	Yu *et al.* 2017^51^	56.51–65.61	0.01–0.012
Mining and smelting	Bi *et al.* 2006^53^	71.0–240.0	0.004–0.056
Municipal wastewater irrigated	Liu *et al.* 2005^57^	49.0–162.0	0.04–0.08
	Asgari and Cornelis 2015^56^	23.8–45.3	0.01–0.12
	Lu *et al.* 2015^62^	11.12–12.05	0.45–1.03
	Zojaji *et al.* 2014^66^	11.15–26.68	0.11–0.40

**Table 11:** Zinc Concentrations in Soil and Bioaccumulation Factors in Zea mays Based on Site Type

**Type of site**	**Reference**	**Zinc (mg kg^−1^)**	**Bioaccumulation factor**
Dumpsite	Oladejo *et al.* 2017^41^	156.78–243.81	0.047–0.4
	Awokunmi *et al.* 2014^40^	63.0–80.2	0.07–0.4
Industrial pollution and inorganic fertilizer	Yu *et al.* 2017^51^	70.58–85.15	0.203–0.245
	Ibrahim *et al.* 2015^59^	27.21–30.78	0.23–0.99
	Nan *et al.* 2002^52^	43.5–565.0	0.189–0.73
Mining and smelting	Bi *et al.* 2006^53^	260.0–5500.0	0.03–0.25
	Rattan *et al.* 2005^63^	99.68–99.86	0.678–0.789
Municipal wastewater irrigated	Liu *et al.* 2005^57^	16.0–162.5	0.44–0.88
	Asgari and Cornelis 2015^56^	146.1–238.9	0.18–0.34
	Lu *et al.* 2015^61^	8.47–12.13	0.1–0.26

**Table 12:** Copper Concentrations in Soil and Bioaccumulation Factors in Zea mays Based on Site Type

**Type of site**	**Reference**	**Copper (mg kg^−1^)**	**Bioaccumulation factor**
Dumpsite	Oladejo *et al.* 2017^41^	20.17–21.41	0.1–0.47
	Awokunmi *et al.* 2014^40^	7.0–18.0	0.44–0.68
Industrial pollution and inorganic fertilizer	Malomo *et al.* 2013^61^	4.0–12.2	0.65–0.94
	Yu *et al.* 2017^51^	19.21–22.63	0.056–0.066
	Ibrahim *et al.* 2015^59^	2.25–33.97	0.011–0.99
Mining and smelting	Bi *et al.* 2006^53^	9.3–260.0	0.015–0.16
Sewage irrigated	Rattan *et al.* 2005^65^	99.91–99.94	0.133–0.149
	Liu *et al.* 2005^57^	13.5–88.0	0.27–0.86
Municipal wastewater irrigated	Asgari and Cornelis 2015^56^	51.9–64.3	0.06–0.12

Table 13 shows the concentration of the selected heavy metals in Zea mays from various research data.

**Table 13:** Concentrations of Cadmium, Lead, Chromium, Zinc, and Copper in Zea mays

**Reference**	**Number of samples**	**Cadmium (mg kg^−1^)**	**Lead (mg kg^−1^)**	**Chromium (mg kg ^−1^)**	**Zinc (mg kg ^−1^)**	**Copper (mg kg^−1^)**
Awokunmi *et al.* 2014^40^	40	4.331	40.2	30.231	18.245	7.66
Oladejo *et al.* 2017^41^	12	0.805	1.433	2.809	52.447	6.04
Ibrahim *et al.* 2015^59^	9	-	20.615	-	18.365	16.8
Mantovia *et al.* 2005^60^	12	-	0.103	0.826	34.265	2.091
Stanislawska–Glubiak *et al.* 2015^42^	16	0.0887	0.33	-	41.55	-
Prabpai *et al.* 2009^43^	20	0.102	0.715	0.128	-	27.32
Mu *et al.* 2013^63^	18	-	-	-	22.217	20.576
Allushllari *et al.* 2013 64	15	-	-	-	30.81	5.012
Yang *et al.* 2013^44^	17	0.037	-	-	-	-
Cai *et al.* 2014^45^	27	0.00764	0.848	1.359	-	-
Li *et al.* 2008^46^	30	0.608	-	0.0826	-	-
Jin *et al.* 2014^47^	27	0.00535	0.667	-	-	-
Zhu 2013^48^	16	0.0066	0.0309	0.286	-	-
Kang *et al.* 2011^49^	15	0.00471	0.00000592	0.334	-	-
Wang *et al.* 2008^50^	12	1.7336	0.186	0.741	-	-
Yu *et al.* 2017^51^	55	0.0183	0.0349	0.676	17.595	1.285
Malomo *et al.* 2013^61^	24	-	110.108	-	-	7.034
Nan *et al.* 2002^52^	33	9.172	-	-	210.335	-
Bi *et al.* 2006^53^	15	20.009	33.975	6.862	691.4	20.87
Rattan *et al.* 2005^65^	115	-	-	-	73.187	14.089
Bi *et al.* 2009^54^	55	-	25.305	-	-	-
Nwite and Alu 2015^55^	27	0.0299	0.184	-	-	-
Lu *et al.* 2015^62^	40	-	1.776	8.708	2.001	-
Zojaji *et al.* 2014^66^	12	-	-	5.949	-	-
Asgari and Cornelis 2015^56^	96	0.669	-	2.837	53.762	5.415
Liu *et al.* 2005^57^	24	0.12	3.238	7.46	75.02	39.663
Afolayan and Hassan 2017^58^	17	39.879	38.114	-	-	-

A one-sample Student's t-test (*Table 14*) was performed to test the hypothesis that the mean concentration of Cd in Zea mays is not significantly different from the permissible limit set by the WHO/Food and Agriculture Organization for Cd in food.^67^ The permissible limit for Cd in all foods is set at 0.1 mg/kg.

**Table 14:** One Sample t-Test for Concentration of Cadmium in Maize

**One-Sample Statistics**						
		N	Mean		Standard deviation	Standard error mean
Cd		18	4.31260		10.184505	2.400511
**One-Sample Test**						
Test value = 0.05						
	t	df	Sig. (2-tailed)	Mean difference	95% Confidence interval of the difference	
					Lower	Upper
Cd	1.755	17	0.097	4.212600	−0.85204	9.27724

Abbreviations: N, sample size; t, test statistic; df, degrees of freedom; sig. (2-tailed), two-tailed p-value corresponding to the test statistic (Supplemental Material 1).

The mean concentrations of Cd in Zea mays (mean = 4.31) was not significantly different (p=0.097) from the WHO value of 0.1 mg/kg, test statistic (t)(17) = 1.76, p=0.09. The confidence interval above is given as −0.85204, 9.27724. Thus, the fact that this interval contains zero indicates that the test would not be rejected at the α = .05 level, and there is not significant evidence that the mean concentration of Cd is different from 0.1 mg/kg. However, Cd is a wellknown heavy metal toxicant with a specific gravity 8.65 times greater than water.

A one-sample Student's t-test (*Table 15*) was performed to test the hypothesis that the mean concentration of Pb in Zea mays is not significantly different from the permissible limit set by WHO for Pb in food. The permissible limit for Pb in all foods is set at 0.2 mg/kg.

**Table 15:** One Sample t-Test for Concentration of Lead in Maize

**One-Sample Statistics**						
		N	Mean		Standard deviation	Standard error mean
Pb		19	14.62436		27.249976	6.251573
**One-Sample Test**						
Test value = 0.2						
	t	df	Sig. (2-tailed)	Mean difference	95% Confidence interval of the difference	
					Lower	Upper
Pb	2.307	18	0.033	14.424361	1.29029	27.55843

Abbreviations: N, sample size; df, degrees of freedom; sig. (2-tailed), two-tailed p-value corresponding to the test statistic (Supplemental Material 2).

The mean concentrations of Pb in Zea mays (mean = 14.62, standard deviation = 27.25, number = 19) were not significantly different from the hypothesized value of 0.2 mg/kg, t(18) = 2.31, p=0.03. The confidence interval above is given as 1.29029, 27.55843. Thus, the fact that this interval does not contain zero indicates that the test would be rejected at the α = .05 level, and that there is significant evidence that the mean concentration of Pb is different from 0.2 mg/kg.

A one-sample Student's t-test (*Table 16*) was performed to test the hypothesis that the mean concentration of Cr in Zea mays is not significantly different from the permissible limit set by WHO for Cr in food. The permissible limit for Cr in all foods is set at 1 mg/kg.

**Table 16:** One Sample t-Test for Concentration of Chromium in Maize

**One-Sample Statistics**						
		N	Mean		Standard deviation	Standard error mean
Cr		15	4.61925		7.688129	1.985066
**One-Sample Test**						
Test value = 1						
	t	df	Sig. (2-tailed)	Mean difference	95% Confidence interval of the difference	
					Lower	Upper
Cr	1.823	14	0.090	3.619253	−0.63829	7.87680

Abbreviations: N, sample size; df, degrees of freedom; sig. (2-tailed), two-tailed p-value corresponding to the test statistic (Supplemental Material 3).

The mean concentrations of Cr in Zea mays (mean = 4.62, standard deviation = 7.69, number = 15) were not significantly different from the hypothesized value of 1 mg/kg, t(14) = 1.82, p=0.09. The confidence interval above is given as −0.63829, 7.87680. Thus, the fact that this interval contains zero indicates that the test would not be rejected at the α = .05 level, and there is not significant evidence that the mean concentration of Cr is different from 1 mg/kg.

A one-sample Student's t-test (*Table 17*) was performed to test the hypothesis that the mean concentration of Cu in Zea mays is not significantly different from the permissible limit set by WHO for Cu in food. The permissible limit for Cu in all foods is set at 10 mg/kg.

**Table 17:** One Sample t-Test for Concentration of Copper in Maize

		N	Mean		Standard deviation	Standard error mean
Cu		13	13.37346		11.325714	3.141188
**One-Sample Test**						
Test value =10						
	t	df	Sig. (2-tailed)	Mean difference	95% Confidence interval of the difference	
					Lower	Upper
Cu	1.074	12	0.304	3.373462	−3.47060	10.21752

Abbreviations: N, sample size; df, degrees of freedom; sig. (2-tailed), two-tailed p-value corresponding to the test statistic (Supplemental Material 4).

The mean concentrations of Cu in Zea mays (mean = 13.37, standard deviation = 11.33, number = 13) were not significantly different from the hypothesized value of 10 mg/kg, t(12) = 1.07, p=0.30. The confidence interval above is given as −3.47060, 10.21752. Thus, the fact that this interval contains zero indicates that the test would not be rejected at the α = .05 level, and there is not significant evidence that the mean concentration of Cu is different from 10 mg/kg.

Copper is an essential element in mammalian nutrition as a component of metallo-enzymes in which it acts as an electron donor or acceptor. Conversely, exposure to high levels of Cu can result in a number of adverse health effects.^70^

## Discussion

Maize is capable of bioaccumulating heavy metals from contaminated soils by translocating them from roots to shoots. Certain metals (e.g. Cd and Pb) have been reportedly accumulated by maize above the level used to define metal hyperaccumulation. Based on its capability of heavy metal uptake and sensitivity to high levels of metal pollution, maize is considered to be an accumulator and a metal tolerant plant, especially for Cd and Zn.^71^ One of the key aspects of the acceptance of phytoextraction pertains to its performance, ultimate utilization of by products and its overall economic viability.

However, the transfer and accumulation of heavy metals from soil to plants is an extremely complex process affected by multiple factors, which exert different influences on the process by means of various mechanisms. Some of the major influencing factors include the chemical forms of the heavy metals, pH of the soil, the soil organic matter content, plant species, climatic conditions, and irrigation with polluted water.^72–77^ As soils in different areas differ greatly in physical and chemical properties, the mechanisms of heavy metal transfer and accumulation in soil-plant systems is complex, and may be the cause for variations in Zea mays accumulation of heavy metals in the literature.^78^ There are a number of factors which influence uptake. Organic acid exudation by plants is a major factor governing the transfer and accumulation of heavy metals as they affect the uptake of heavy metals by altering the rhizosphere processes responsible for nutrient uptake. Flavonoid exudation can influence nutrient cycles by interacting with proteins and making protein nitrogen more resistant to microbial degradation and could indirectly affect soil pH, thus influencing heavy metals activity. Clay minerals and other soil colloids may also influence the bioavailability of heavy metals (a study by Zhou and Li (1996) suggested that for a given soil pH, increasing the proportion of particles with a size smaller than 0.002 mm can increase the soil capacity for Zn adsorption, thereby limiting the transfer of Zn to plants). Microbes are another factor affecting heavy metals uptake by plants.^79–87^ The number of microbes around the roots is higher than that in other parts of the soil. Bacteria and fungi surrounding plant roots can promote plant uptake of heavy metals by changing heavy metals activity.^88^ For example, some bacteria can reduce As, mercury, and selenium, while others can oxidize iron and As.^89^ Among the various mechanisms of plant absorption, passive uptake via micro pores in the cell walls of root cells is the main method by which heavy metals access plants; chemical species with a high redox activity and chelating agents in soil may affect heavy metal uptake and accumulation in plants by changing their chemical form and thus their availability for plants.^90^ Nitrogen in soil represents another important factor influencing the transfer of heavy metals in soil-plant systems. It affects the bioavailability of heavy metals mainly by altering soil acidity.^91–94^ Soil pH is another factor influencing uptake of heavy metals from the soil. Among heavy metals, Cd is the most sensitive to soil pH.^95^,^96^ In addition, ammonium ions can displace trace metals in exchangeable forms or facilitate their release from soil colloids.^97^ The changes in concentration of soil-free bases and pH resulting from the dissolution and diffusion of ammonia nitrogen in soil in turn may change the solubility of heavy metals in soil.^98^ The mobility of heavy metals in soil-plant systems is also affected by the way heavy metals enter the plants. Chemical elements are primarily uptaken by roots from the soil or by leaves from the atmosphere. Apart from differences among various plant species and cultivars, the overall combination of soil physical and chemical properties controls both the rate and extent of metal uptake.^99^ For instance, a small decrease in biomass yield was observed in the case of maize plants grown on sandy soil, whereas in plants grown on loamy soil a significant increase in plant yields and decrease in Cu concentrations in shoot biomass were observed.^100^ The ideal soil types for maize plants are loam or silt loam surface soil and brown silt clay loam with a fairly permeable sub soil.^101^ The time period of growth can significantly affect the biomass yield of maize.

The degradation and depletion of the soil environment are a consequence of human activities such as deposition and discharge of agricultural residues on lands and water bodies and increased use of fertilizers and pesticides. The WHO reported that normal concentrations of Pb in soil range from 15–30 mg/kg, although from most of the reviewed literature, Pb values were much higher than this value.^102^ The allowed amount of Cd and Pb in fertilizer ranges from Cd = 8–300 mg kg^−1^, and Pb = 20–200 mg kg^−1^. These values vary among countries, and thus there is no specific fertilizer law.^103^ Clear guidelines or permissible limits for heavy metals have not been published by any regulatory body such as the United States Environmental Protection Agency (USEPA), the WHO or the Food and Agriculture Organization, probably due to variation in soil types, especially soil pH which affects bioavailability of heavy metals, and varying plant types. However, there are specific permissible limits based on soil type in different regions of the world. In 2005, the Ministry of Environmental Protection of the People's Republic of China (now the Ministry of Ecology and Environment) set permissible limits of 0.6 mg/kg, 100 mg/kg, 350 mg/kg, 300 mg/kg, 250 mg/kg for Cd, Cu, Pb, Zn and Cr, respectively.^104^ A group of Dutch ecologists set limits of 0.76 mg/kg for Cd, 3.6 mg/kg for Cr, 3.5 mg/kg for Cu, 16 mg/kg for Zn, and 55 mg/kg for Pb. 105 The European Commission also set 1.5 mg/kg, 100 mg/kg, 100 mg/kg, and 200 mg/kg for Cd, Cu, Pb and Zn, respectively.^106^ The concentration of heavy metals in soils of reviewed studies were higher than the maximum tolerable levels proposed for agricultural soils.

Statistical analysis of the available data shows that there is a significant difference between mean concentrations of Pb and the maximum level set by the Food and Agriculture Organization of the World Health Organization (FAO/WHO).^67^ There was no significant difference between the mean concentrations of Cd, Cr, and Cu from the analyzed data and the standard set by WHO. However, the presence of these heavy metals in food is of great health risk concern. Zinc was evaluated by the Joint FAO/WHO Expert Committee on Food Additives in 1966 and 1982 based on clinical studies in which up to 600 mg of Zn sulfate (equivalent to 200 mg elemental Zn) was administered daily in divided doses for a period of several months, without any reported adverse effects, including effects on blood counts and serum biochemistry.^67^ There is a wide margin between nutritionally required amounts of Zn and toxic levels. Taking into account recent studies on humans, the WHO proposed in 2003 that the derivation of a guideline value was not required at the time; it was stated however, that drinking water containing Zn at levels above 3 mg/liter may not be acceptable to consumers based on taste considerations.^67^

Tables 6–10 show previously published research on Zea mays planted on heavy metal polluted sites. Cadmium and Cr concentrations in soil were the highest in a tin-mining area, where long-term mining, transportation, mineral processing, and other production activities caused heavy metal pollution in the soil of local farmlands.^53^,^54^ The soil at a battery waste dumpsite exhibited the highest Pb concentration, while the soil at a Zn smelting site presented the highest level of Zn.^58^ The heavy metals contained in wastes at these dumps can enter and contaminate the soil via long-term leaching and infiltration.^68^,^69^ The highest soil Cu concentration was found in mining and smelting facility.^53^ Awokunmi *et al*. and Oladejo *et al*. reported high levels of heavy metal accumulation in dumpsites in Nigeria.^40^,^41^ They revealed that dumpsites are sinks for elevated levels of heavy metals. Their work showed accumulation of Pb, Cd, Cr, Cu and Zn in Zea mays planted on these heavy metal concentrated soils. They also found that all of the heavy metals studied were found to accumulate mainly in the roots of the maize plant. Zea mays L. proves to be heavy metal tolerant and has high metal accumulating ability in the foliar parts with moderate BAF.

The bioconcentration factors (BCF) of Cd, Zn, Pb, Cr, and Cu in Zea mays generally increased with increasing heavy metal concentrations in the soil. The BCF of Cd, Zn, and Cu were high, while Cu and Cd easily accumulate in maize plants. These findings suggest that crops with high capacities for accumulating Cd, Cu, and Zn should be avoided during crop selection in order to reduce the risks to human health posed by the presence of heavy metals in crops. An analysis of the available data on Zea mays revealed that the concentrations of Cd, Cr, Pb, Cu, and Zn are high in Zea mays, but the statistical analysis identified many factors affecting the uptake of heavy metals from the soil, beyond the concentration of heavy metal in the soil. Studies have indicated that Pb concentration in exposed plants increased as the concentration in the soil increased. The results of the present study affirmed maize as a significant accumulator of Pb and Cd.

In general, Pb bioaccumulation was higher in the root tissues compared with the shoot tissues.^40^,^41^,^107^,^108^ This trend was also observed in grass species (Agrogyron elongatum) and monocotyledon salt marsh plants.^109^,^110^ Plants are considered to be hyperaccumulators when they actively take up exceedingly large amounts of one or more heavy metals from the soil.^110^ Hyperaccumulators accumulate heavy metals in their shoot 100–1000 times higher than nonaccumulators.^112^ Reports have also shown that Cd-hyperaccumulating plant species in general accumulate more than 1 mg/kg of Cd, whereas regular plants accumulate only about 0.001–0.05 mg/kg of Cd.^113^ Zea mays has been observed to accumulate Cd in excess of this level.^40^,^54^,^58^ In the literature review, Zea mays accumulated above normal levels on many occasions, but on some occasions did not accumulate above normal levels. This may be due to various factors including soil heavy metal concentration and other influencing factors, such as the chemical forms of the heavy metals, soil pH, the soil organic matter content, plant species, and climatic conditions.^72–76^ It was noted that Pb bioaccumulation of corn in the root tissues at the highest treatment level were comparable with the Pb bioaccumulation values of Brassica juncea, which is a well-known hyperaccumulator species.^107^ These results further indicate that the Pb bioaccumulation capability of Zea mays is on par with other hyperaccumulating plants. The increase in accumulation and uptake of Pb in the root tissues could be attributed to their proximity to the Pb source. This contention was supported by other studies which showed that high concentrations of Pb available near the root system lead to an increase in Pb uptake and accumulation.^114^,^115^ Studies have shown that greater bioaccumulations of Pb in the shoot tissue of seedling at treatments of 100, 500, 2000 and 5000 mg/kg correlates with increasing concentration of Pb in soil.^107^ Lead taken up by the roots is transported and precipitated throughout the plant corresponding with the presence of Pb in the shoot tissues, and the connection of conducting vascular tissues of corn could be the main pathway of Pb uptake from the root to the shoot area.^116^ Likewise, comparing the bioaccumulation in Zea mays with Raphanus sativa revealed that the former accumulated more Pb compared to the latter, indicating that maize has a greater tolerance to Pb or higher tendency to accumulate Pb.^107^,^114^ This suggests that Zea mays has the ability to accumulate considerable amounts of Pb, and is thus a potential candidate for remediating Pb-contaminated soils.

Some studies showed accumulation of Pb, Cd, Cr, Cu and Zn in Zea mays planted on heavy metal concentrated soils.^40^,^41^They also revealed that all of the studied heavy metals were found to accumulate mainly in the roots of the maize plant. Chromium concentration in the roots was reported to be higher than other parts.^66^ The present study found that Cr was mainly immobilized in the roots in comparison with other parts of Zea mays. Studies on vegetables also supported this point.^118^,^119^ This study clearly demonstrated that maize accumulated high amounts of Pb from the soil. These findings show the potential of Zea mays to ameliorate Pb-contaminated soil. Identification of tolerant plant species growing in heavy metal contaminated sites and characterization of their Pb bioaccumulation properties are essential for the proper use and management of contaminated areas. Heavy metals accumulation decreased in the following order: soil>root>leaves>grains. However, the stalk and stem of plants show higher tendencies of metal accumulation compared to maize silk and grain. Other plant parts such as stem, stalk and silk were also found to contain these metals, indicating that they are unsafe for use as animal feedstock.

The current study revealed that dumpsites are sinks for elevated levels of heavy metals. It is important to note that if peasant farmers continue to cultivate maize and other arable crops on abandoned dumpsites, this will increase the levels of heavy metals which may eventually enter the food chain and present possible adverse human health effects. Heavy metal accumulation in soils and plants is of increasing concern due to the potential human health risks. Heavy metal contaminants not only represent a threat to agricultural product safety, but also harm to immune, reproductive, and nervous systems of organisms after entering their bodies through ingestion. This eventually leads to food chain contamination, which is an important pathway for entry of toxic pollutants into the human body. Heavy metals uptake by plants and successive accumulation in human tissues and biomagnifications through the food chain causes both human health and environment concerns. Since the toxic heavy metal concentrations in the most commonly consumed parts of the maize plant are high, there is a tendency for these metals to transfer to both animals (through leaves and stem used as fodder) and humans, presenting human health risks. Thus, as maize plants grown on the topsoil of waste dumpsites have shown active phytoextraction via the root as well as high bioaccumulative properties, this study recommends that the maize grown near dumpsites is unfit for human and animal consumption. These crops could expose residents and livestock to the undesired effects of heavy metal toxicity.

## Conclusions

An analysis of the available data on Zea mays revealed that the concentrations of Cd, Cr, Pb, Cu, and Zn are high in Zea mays. Metals uptake by plants from soil reduces crop productivity by inhibiting physiological metabolism. Accumulation of heavy metals in plants and biomagnification presents a threat to human and environmental health. Excessive accumulation of heavy metals in agricultural soils often leads to elevated heavy metal uptake by crops, and thus affects food quality and safety.^120^ Food chain contamination is an important pathway for the entry of toxic pollutants into the human body. Heavy metals have been proven to be toxic to both humans and the environment. Therefore, regular environmental monitoring and restoration is imperative to determine that soils used for maize farming are not contaminated with heavy metals.

Owing to their toxicity and possible bioaccumulation, these compounds should be subject to mandatory monitoring. Governments should promote harmonized data collection, research, legislation and regulations, and consider the use of indicators. Assessments determining the chemical concentration scenario and the use of biomarkers should provide useful data to set standards and guideline values designed to protect human and environmental health from heavy metal contaminants. Exposure measurements are essential for the protection of high-risk populations and subgroups. Furthermore, governments should, when setting acceptable levels or criteria related to chemicals, take into consideration the potential enhanced exposures and/or vulnerabilities of children.

The ability of Zea mays to accumulate heavy metals from soil is significantly affected by a number of factors aside from the concentration of heavy metals in soil. However, based on the outcomes of the present study, it was concluded that Zea mays is capable of accumulating all selected heavy metals from soil over a wide range of environmental conditions.
