# Supplementary material for: Bioaccumulation Factor of Selected Heavy Metals in Zea mays
Source: J Health Pollut. 2019 Dec 6;9(24):191207. doi: 10.5696/2156-9614-9.24.191207 (PMC6905145; doi:10.5696/2156-9614-9.24.191207)
Supplement: Supplementary file 1 [file Aladesanmi_Supplemental_Material.docx]

**Supplemental Material**

| **Case processing summary** | | | | | | | | | |  |
| --- | --- | --- | --- | --- | --- | --- | --- | --- | --- | --- |
|  | **Cases** | | | | | | | | |  |
|  | **Valid** | | **Missing** | | | | **Total** | | |  |
|  | **N** | **Percent** | **N** | | **Percent** | | **N** | | **Percent** |  |
| Cd | 18 | 94.7% | 1 | | 5.3% | | 19 | | 100.0% |  |
| Pb | 19 | 100.0% | 0 | | 0.0% | | 19 | | 100.0% |  |
| Cr | 15 | 78.9% | 4 | | 21.1% | | 19 | | 100.0% |  |
| Zn | 14 | 73.7% | 5 | | 26.3% | | 19 | | 100.0% |  |
| Cu | 13 | 68.4% | 6 | | 31.6% | | 19 | | 100.0% |  |
| **Supplemental Material 1** | | | | | | | | | | |
| **Descriptives** | | | | | | | | | | |
|  | | | | | | **Statistic** | | **Standard error** | | |
| Cd | Mean | | | | | 4.31260 | | 2.400511 | | |
|  | 95% Confidence interval for mean | | | Lower bound | | -0.75204 | |  | | |
|  |  |  |  | Upper bound | | 9.37724 | |  | | |
|  | 5% Trimmed mean | | | | | 2.57602 | |  | | |
|  | Median | | | | | 0.11100 | |  | | |
|  | Variance | | | | | 103.724 | |  | | |
|  | Standard deviation | | | | | 10.184505 | |  | | |
|  | Minimum | | | | | 0.005 | |  | | |
|  | Maximum | | | | | 39.879 | |  | | |
|  | Range | | | | | 39.874 | |  | | |
|  | Interquartile range | | | | | 2.367 | |  | | |
|  | Skewness | | | | | 3.009 | | 0.536 | | |
|  | Kurtosis | | | | | 9.362 | | 1.038 | | |
| **Supplemental Material 2** | | | | | | | | | | |
| **Descriptives** | | | | | | | | | | |
|  | | | | | | **Statistic** | | **Standard error** | | |
| Pb | Mean | | | | | 14.62436 | | 6.251573 | | |
|  | 95% Confidence interval for mean | | | Lower bound | | 1.49029 | |  | | |
|  |  |  |  | Upper bound | | 27.75843 | |  | | |
|  | 5% Trimmed mean | | | | | 10.13218 | |  | | |
|  | Median | | | | | 0.84800 | |  | | |
|  | Variance | | | | | 742.561 | |  | | |
|  | Standard deviation | | | | | 27.249976 | |  | | |
|  | Minimum | | | | | 0.000 | |  | | |
|  | Maximum | | | | | 110.108 | |  | | |
|  | Range | | | | | 110.108 | |  | | |
|  | Interquartile range | | | | | 25.121 | |  | | |
|  | Skewness | | | | | 2.681 | | 0.524 | | |
|  | Kurtosis | | | | | 8.321 | | 1.014 | | |

| **Supplemental Material 3** | | | | |
| --- | --- | --- | --- | --- |
| **Descriptives** | | | | |
|  | | | **Statistic** | **Standard error** |
| Cr | Mean | | 4.61925 | 1.985066 |
|  | 95% Confidence interval for mean | Lower bound | 0.36171 |  |
|  |  | Upper bound | 8.87680 |  |
|  | 5% Trimmed mean | | 3.44841 |  |
|  | Median | | 1.35900 |  |
|  | Variance | | 59.107 |  |
|  | Standard deviation | | 7.688129 |  |
|  | Minimum | | 0.083 |  |
|  | Maximum | | 30.231 |  |
|  | Range | | 30.148 |  |
|  | Interquartile range | | 6.528 |  |
|  | Skewness | | 2.964 | 0.580 |
|  | Kurtosis | | 9.850 | 1.121 |

**Supplemental Material 4**

| **Descriptives** | | | | |
| --- | --- | --- | --- | --- |
|  | | | **Statistic** | **Standard error** |
| Cu | Mean | | 13.37346 | 3.141188 |
|  | 95% Confidence interval for mean | Lower bound | 6.52940 |  |
|  |  | Upper bound | 20.21752 |  |
|  | 5% Trimmed mean | | 12.58451 |  |
|  | Median | | 7.66000 |  |
|  | Variance | | 128.272 |  |
|  | Standard deviation | | 11.325714 |  |
|  | Minimum | | 1.285 |  |
|  | Maximum | | 39.663 |  |
|  | Range | | 38.378 |  |
|  | Interquartile range | | 15.510 |  |
|  | Skewness | | 1.125 | 0.616 |
|  | Kurtosis | | 0.879 | 1.191 |
